# Lethal and sublethal effects of toxicants on bumble bee populations: a modelling approach

**DOI:** 10.1007/s10646-020-02162-y

**Published:** 2020-02-14

**Authors:** J. E. Banks, H. T. Banks, N. Myers, A. N. Laubmeier, R. Bommarco

**Affiliations:** 1 Undergraduate Research Opportunities Center, California State University, Monterey Bay, Seaside, CA 93955 USA; 2 Center for Research in Scientific Computation, North Carolina State University, Raleigh, NC 27695-8212 USA; 3 Department of Ecology, Swedish University of Agricultural Sciences, Uppsala, 750 07 Sweden; 4 Present Address: Department of Mathematics, University of Nebraska-Lincoln, Lincoln, 68588–0130 NE USA

**Keywords:** Hymenoptera, Neonicitinoid, Delay differential equation

## Abstract

Pollinator decline worldwide is well-documented; globally, chemical pesticides (especially the class of pesticides known as neonicotinoids) have been implicated in hymenopteran decline, but the mechanics and drivers of population trends and dynamics of wild bees is poorly understood. Declines and shifts in community composition of bumble bees (Bombus *spp*.) have been documented in North America and Europe, with a suite of lethal and sub-lethal effects of pesticides on bumble bee populations documented. We employ a mathematical model parameterized with values taken from the literature that uses differential equations to track bumble bee populations through time in order to attain a better understanding of toxicant effects on a developing colony of bumble bees. We use a delay differential equation (DDE) model, which requires fewer parameter estimations than agent-based models while affording us the ability to explicitly describe the effect of larval incubation and colony history on population outcomes. We explore how both lethal and sublethal effects such as reduced foraging ability may combine to affect population outcomes, and discuss the implications for the protection and conservation of ecosystem services.

## Introduction

The protection of ecosystem services has become a major focus of applied ecology, with one emphasis on understanding population processes of pollinators and biological control agents. Pollinator conservation in particular has received much attention due to their well-documented decline coupled with their ability to significantly contribute to crop pollination (Klein et al. 2007; Wratten et al. 2012). Globally, chemical pesticides (especially the class known as neonicotinoids) have been implicated in hymenopteran decline (Desneux et al. 2007; Goulson et al 2015; Lundin et al. 2015; Rundlöf et al. 2015). Exposure to pesticides has been implicated in deficits in both short- and long-term learning as well as memory and sensory capabilities, all of which can affect foraging efficiency and provisioning (Tan et al. 2015; Klein et al. 2017). Within-colony behavior related to caretaking, which can have implications for thermoregulation and colony survival, may also be affected by pesticide exposure (Crall et al. 2018). Despite our increased understanding of the effects of pesticide exposure on bee physiology and behavior, the overall effects of pesticides on population dynamics of bees remain poorly understood. Furthermore, much of what we do know about population processes of pollinators stems from work conducted with honeybees (*Apis melifera*); recent simulation models have identified the potential for sublethal effects on honeybees stemming from varroa mites and other stressors (Becher et al. 2014; Thorbek et al. 2017), while other models have underscored the complex relationships between food availability and honeybee foraging and survival (Khoury et al. 2013; Perry et al. 2015).

More recently, attention has increasingly focused on non-*Apis* bees, especially wild bees. In particular, declines and shifts in community composition of bumble bees (Bombus *spp*.) have been documented in North America and Europe (Biesmeijer et al. 2006; Bommarco et al. 2011; Bartomeus et al. 2013). A suite of lethal and sub-lethal effects of pesticides on bumble bee populations have been demonstrated, including reductions in foraging ability and other behavioral changes (Brittain and Potts 2011; Feltham et al. 2014; Barbosa et al. 2015; Stanley et al. 2016; Switzer and Combes 2016; Phelps et al. 2018; Lämsä et al. 2018). A population-level perspective is critical in linking what we know about individual toxicant effects to the long-term effects of pesticide exposure on bumble bee populations.

Pesticide risk assessment in the United States for all arthropods is based on acute toxicity tests (LC_50_) on a single species—the European honeybee (*A. mellifera*)—making that organism an ideal starting point for understanding the effects of chemical stressors for other bees. However, we have shown that, due to subtle differences in life histories, even closely related hymenopteran species can exhibit markedly different population responses to the same toxic insults (Banks et al. 2011; Banks et al. 2014). Further complicating matters, work done at the physiological level reveals that different bee species exhibit different levels of susceptibility to the same chemical pesticides (Manjon et al. 2018). Taken together, what we know about the effects of toxicants on one species (e.g., honeybees) does not necessarily translate to a good understanding of the effects of toxicants on other even closely related species such as bumble bees; responses to toxicants need to be evaluated for each species. Furthermore, it is now well established that acute tests such as LC/LD_50_, historically the gold standard for comparing toxicological effects, fail to capture longer-term population outcomes (including sublethal effects) and could be woefully misleading (Banks and Stark 1998; Stark and Banks 2003; Stark et al. 2004; Desneux et al. 2007; Forbes et al. 2011; Biondi et al. 2013; Stark et al. 2015). Finally, most studies of chemical toxicity related to bumble bees have focused on a single toxicant or pesticide, when in practice in the field bees are subjected to multiple toxicants acting in both lethal and sublethal ways (Stark et al. 2007). Here we seek a better understanding of toxicant effects on a developing colony of bumble bees over time, as well as insights into how acute and sublethal effects (either from the same or different chemical toxicants) may combine to affect population outcomes.

The utilization of computational models in bumble bee research has increased in recent years although it still has not been as exhaustive as efforts on honeybees. Many models have focused on foraging dynamics by workers as they influence different metrics of colony growth (Oster 1976; Olsson et al. 2015; Crone and Williams 2016; Häussler et al. 2017). Becher et al. (2018) used agent-based modeling to understand hive dynamics, examining the influence of pesticides on multi-generational colony dynamics, though they did not explore effects on colony interior dynamics. Other similar models indicate that pesticides and other stressors can impact colony dynamics, for example by impairing worker bee productivity (Bryden et al. 2013) or queen fecundity (Cresswell 2017). These studies rely on differential and difference equations, in which changes to the colony at any time depend on the current state of the colony. However, changes to a colony might also depend on prior states of the colony, for example due to the length of larval incubation or history of resource availability. We describe these dependencies with a delay differential equation model, parameterized with values taken from the literature.

## Methods and materials

We modelled a single colony of bumble bees using a non-linear system of delay differential equations (DDE) that describe twelve state variables: in-nest nectar abundance *N*(*t*), in-nest pollen abundance *P*(*t*), workers *W*(*t*) and their larvae (modeled as a two-stage population, L^(w)^_1_, L^(w)^_2_), males and their larvae (modeled as a two-stage population, (L^(m)^_1_, L^(m)^_2_), and gynes (new queens) and their larvae (modeled as a three-stage population, (L^(g)^_1_, L^(g)^_2_), L^(g)^_3_).

The model describes the development of the reproductive classes by means of critical colony functions such as resource management, worker caregiving, and population control. It utilizes larval development as the link between colony resources and the adult bumble bee members. Parameter values were drawn from published studies on *Bombus terrestris* and the model was simulated by a direct application of MATLAB delay differential equation solver, *dde223*, to the mathematical model (MATLAB 2016a; Shampine and Thompson 2001). All parameters (Table 1) and full mathematical model (Appendix I) are provided.

**Table 1:** Model variables and parameters. The selected value for simulations and attributions are given in the last column

Variable	Description	Units	Estimate
*t*	Time	Days	
*N*(*t*)	Amount of in-nest nectar	ml	
*P*(*t*)	Amount of in-nest pollen	g	
*W*(*t*)	Number of workers	Individuals (workers)	
$L_1^{\left( w \right)}\left( t \right),\,L_2^{\left( w \right)}\left( t \right)$	Number of worker larvae	Individuals (larvae)	
*M*(*t*)	Number of males	Individuals (males)	
$L_1^{\left( m \right)}\left( t \right),\,L_2^{\left( m \right)}\left( t \right)$	Number of male larvae	Individuals (larvae)	
*G*(*t*)	Number of gynes	Individuals (gynes)	
$L_1^{\left( g \right)}\left( t \right),\,L_2^{\left( g \right)}\left( t \right),\,L_3^{\left( g \right)}\left( t \right)$	Number of gynes larvae	Individuals (larvae)	
**Timeline**			
*T*_*s*_	First day of spring		0
*T*_*s*_ + 22	First workers emerge		22 (Duchateau and Velthuis 1988)
T^*^	First day male/gynes eggs laid		40 (Müller et al. 1992)
T^**^	End of worker eggs laid		44 (Müller et al. 1992)
*T*_*W*_	Beginning of winter		120
**Parameters**			
*b*_*NW*_	Worker nectar collection rate	$\frac{{{\mathrm{ml}}}}{{{\mathrm{day}} \ast {\mathrm{individual}}\,\left( {\mathrm{W}} \right)}}$	0.6 (Goulson et al. 2002; Peat Goulson 2005)
*b*_*PW*_	Worker pollen collection rate	$\frac{{\mathrm{g}}}{{{\mathrm{day}} \ast {\mathrm{individual}}\,\left( {\mathrm{W}} \right)}}$	0.4 (Goulson et al. 2002; Feltham et al. 2014)
*μ*_*NW*_	Worker nectar consumption rate	$\frac{{{\mathrm{ml}}}}{{{\mathrm{day}} \ast {\mathrm{individual}}\,\left( {\mathrm{W}} \right)}}$	0.35 (Tasei et al. 2000)
*μ*_*PW*_	Worker pollen consumption rate	$\frac{g}{{{\mathrm{day}} \ast {\mathrm{individual}}\,\left( {\mathrm{W}} \right)}}$	0.25 (Tasei et al. 2000)
*c*_*i*_	Larval pollen consumption rates	$\frac{g}{{{\mathrm{day}} \ast {\mathrm{individual}}\,\left( {\mathrm{L}} \right)}}$	(0.01, 0.25) (Ribeiro 1994)
*b*_*W*_(*t*)	Worker birth rate	$\frac{{{\mathrm{workers}}}}{{{\mathrm{day}}}}$	8.5 (Duchateau and Velthuis 1988)
*b*_*M*_(*t*)	Male birth rate	$\frac{{{\mathrm{males}}}}{{{\mathrm{day}}}}$	2 (Duchateau and Velthuis 1988)
*b*_*G*_(*t*)	Gyne birth rate	$\frac{{{\mathrm{gynes}}}}{{{\mathrm{day}}}}$	2.6 (Duchateau and Velthuis 1988)
*μ*_*W*_	Worker death rate	$\frac{1}{{{\mathrm{day}}}}$	0.05
*Z*	Larvae to worker ratio		4 (Duchateau and Velthuis 1988)
*α*	Max ejection rate (negligence)	$\frac{{{\mathrm{individual}}\,\left( {\mathrm{L}} \right)}}{{{\mathrm{day}} \ast {\mathrm{individual}}\,\left( {\mathrm{W}} \right)}}$	0.75
*β*	Max ejection rate (malnutrition)	$\frac{{{\mathrm{individual}}\,\left( {\mathrm{L}} \right)}}{{{\mathrm{day}} \ast {\mathrm{individual}}\,\left( {\mathrm{W}} \right)}}$	0.75
*∈*	Roundoff correction factor		0.001

The time frame for the simulated colony begins 22 days after hive initiation in the spring, where day 0 is the first day of spring, *T*_s_, when the first brood of workers emerge to begin gathering nectar and pollen as well as larval feeding and ejection (if necessary) until the beginning of winter when hive functions cease. The switch time, which represents the time when a colony changes from producing worker offspring to male and gyne offspring, is a distinguishing event in a colony’s development (Duchateau and Velthuis 1988). We fixed a late switch time at T^*^ = 40, so that male and gyne larvae appear at day 44, coincidentally the same day the last worker eggs are laid. Development times for each larval subclass was assumed to be fixed (see Table 2, Appendix 1). We note that larvae were subdivided into age groups and we assumed that consumption was constant across each age group, with nectar being consumed at twice the rate of pollen (Pereboom 2000). The model we built was a system of delay differential equations (DDE’s) which is appropriate to use in age structured population models (Murdoch et al.,^1987^; Hartung et al. 2006; Banks 2012; Banks et al. 2017). The model included time varying larval mortality rates (*μ*^(*w*)^ (*t*), *μ*^(*m*)^ (*t*), *μ*^(*g*)^ (*t*)) which were based on past values of the workers, pollen, and nectar variables. The DDE system tracks cumulative larval mortality rates through the *Φ*^(*w*)^(*t*), *Φ*^(*m*)^ (*t*), and *Φ*^(*g*)^ (*t*) variables, allowing us to calculate the development of broods independent of each other over a continuous spectrum, something that is not possible with ordinary differential equations. The larval mortality rate represents the rate at which larvae are ejected from the hive per worker based on two conditions: whether or not there are sufficient resources to nourish existing larvae, and whether or not enough workers are present to tend to the larvae (Pomeroy 1979; Genisse et al. 2002; Tasei and Aupinel 2008; Roger et al. 2017). Estimating these values required a comparison between projected pollen consumption (*C* = *c*_1_(L^(w)^_1_ + L^(m)^_1_ + L^(g)^_1_) + *c*_2_ L^(w)^_2_ + *c*_3_ L^(m)^_2_ + *c*_4_ L^(g)^_2_ + *c*_5_ L^(g)^_3_) and the available pollen P(t) at that time, where *c*_1_ to *c*_5_ are larval pollen consumption rates (a similar comparison was made for nectar as well.) We made a similar comparison between the number of larvae requiring care (L^(w)^_1_ + …. + L^(g)^_3_) to the number of larvae the worker population can support (ZW, where *Z* is the number of larvae a single worker can optimally care for) to determine whether or not proper feeding and care could be provided to the existing larvae by the available workers within the colony (Pomeroy 1979; Tasei et al. 2000); see Appendix 1 for more detail. The other form of population regulation within a hive was exhibited through oophagy, or the consumption of eggs by the worker or queen. Although this behavior is not strictly a population control measure, it can be a significant behavior when malnourishment occurs in the hive (Genissel et al. 2002). The degree of oophagy was calculated using a comparison between desired resource consumption and available resources, in the same way that larval ejection was calculated; values were then incorporated directly into egg laying rates in the model *b*_W_^*^(*t*), *b*_M_^*^(*t*), and *b*_G_^*^(*t*). These functions represent the number of eggs laid at the time *t* which will become larvae. Overall, these mechanisms yielded model expressions such as *b*_W_^*^(*t*−22) exp[*Φ*^(*W*)^ (*t* − 18) − *Φ*^(*W*)^ (*t* − 9)], which represented new workers on day *t* whose eggs were laid 22 days prior, having begun the larval phase 18 days before and survived to begin pupation 9 days previously.

**Table 2:** Fixed duration (in days) of bumble bee life stages in model

Class	Egg	$L_1^k$	$L_2^k$	$L_3^k$	Pupa	Total age
Worker	4	6	3	–	9	22
Male	4	8	3	–	11	26
Gyne	4	5	4	4	13	30

We used the model to simulate toxicant effects in different scenarios that reflect documented impacts of pesticide exposure in the literature. In particular, we simulated (i) lethal direct effects on workers, (ii) sublethal effects via reduced foraging abilities and reduced brood sizes, (iii) combination of lethal and sublethal effects together (Feltham et al. 2014; Laycock et al. 2012; Laycock et al. 2014). We ran simulations for each of these situations measuring the cumulative reproductive output (males and gynes) as our primary metric of population effect. Initially the model was parameterized using values associated with *Bombus terrestris*, although we acknowledge that the model can accommodate other species of bumble bees with appropriate parameter values. To simulate acute effects of pesticide exposure, adult worker populations were culled at the time of exposure to the LD_50_. Exposure did not extend past the day it was introduced to the population. Next, we simulated the sublethal effect of reduced foraging ability, thereby reducing pollen and nectar resources available to the colony and measuring the resulting reproductive output. These effects were modeled by directly impacting the rates that adult workers collect pollen and nectar. We also simulated reductions in the initial broods, corresponding to a sublethal effect on the queen’s egg-laying rate. The egg-laying rate was adjusted appropriately based on whether the first or second brood was affected by the colony’s exposure to the pesticide and the overall reproductive output was measured. Finally, we simulated both lethal and sublethal effects and noted their combined effects on reproductive output.

## Results

### Control

**Fig. 1:**
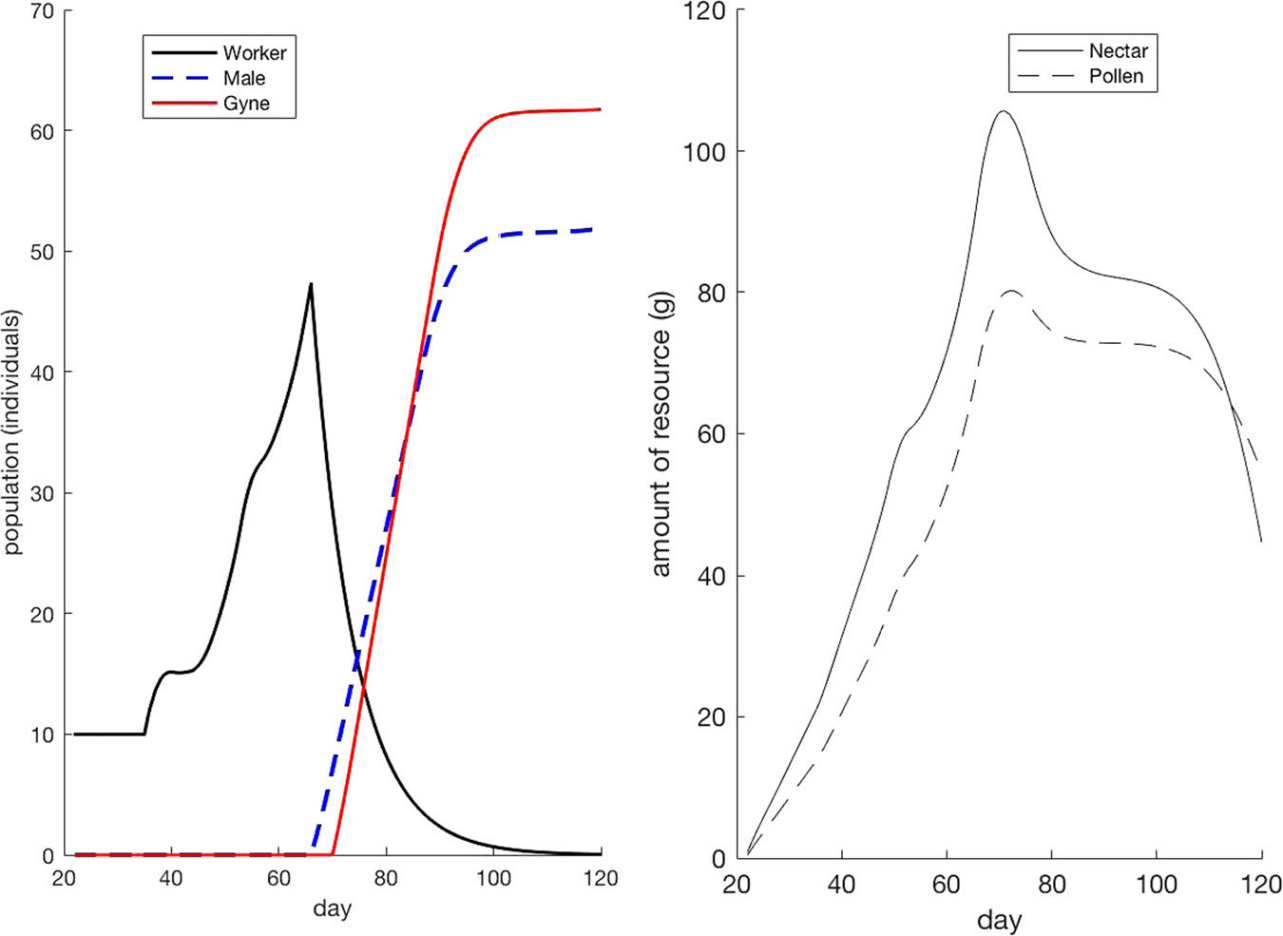
Bumble bee colony simulation over 120 days, including dynamics for both resources, adult workers and cumulative adult reproductive members (males and gynes)

In the absence of toxicological insult, the model produced an increase in the number of workers until around day 60, after which workers declined and males and gynes (reproductives) increased nearly exponentially before plateauing off around 100 days after the start of the simulation. Pollen and nectar resource levels also declined between 70 and 80 days after the simulation, corresponding roughly with the decline in the worker population (Fig. 1).

### Lethal pesticide effects

**Fig. 2:**
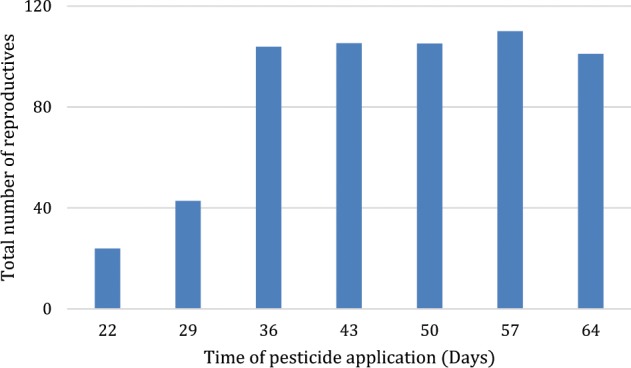
Acute effects of LD_50_ dose on cumulative males and gynes produced in the colony as a function of the timing of pesticide application

Acute pesticide effects were characterized as immediate reductions in the worker population corresponding to the LD_50_ dose of pesticide applied. We varied the time of exposure, noting the impact that delaying contact to pesticide may have on reproductive output. Simulation of an acute effect of pesticides on workers—corresponding to the LD_50_—resulted in a marked decline of reproductive output when exposure to the toxicant occurred during the first 30 days of the simulation. However, results varied as a function of the timing of the exposure; application of the toxicant at 36 days after the start and beyond resulted in much less severe effects (Fig. 2).

### Sublethal pesticide effects

**Fig. 3:**
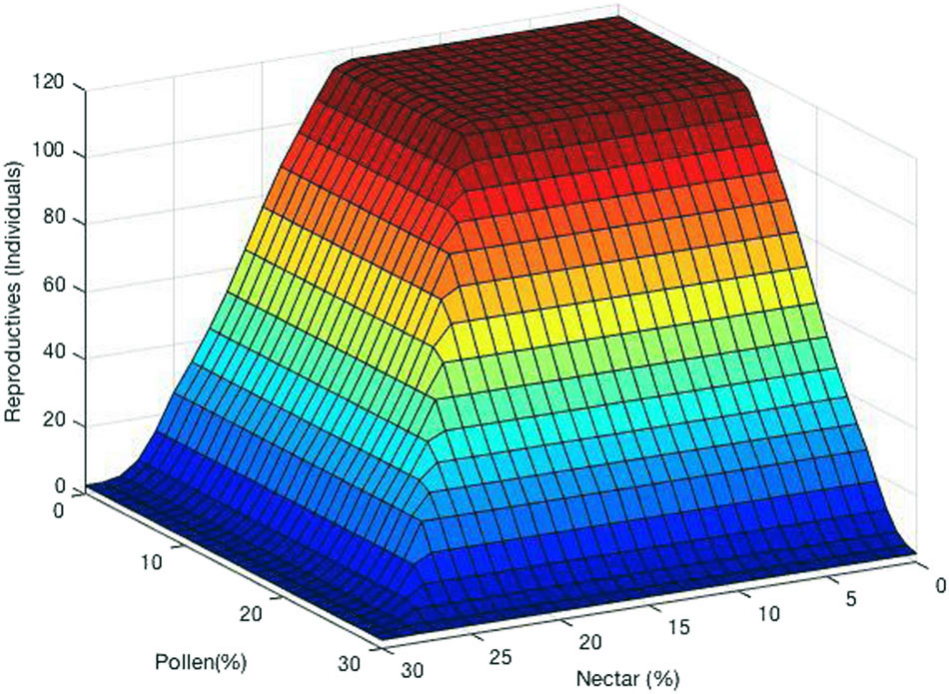
Influence of sublethal effect of reducing foraging ability (by percentage) on bumble bee reproductive output (males + gynes)

**Fig. 4:**
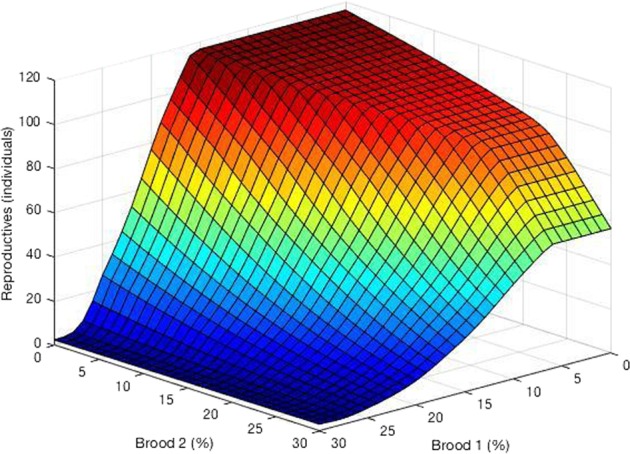
The effect of sublethal reductions to egg laying rates (on 1st and 2nd broods) on cumulative reproductive output of the colony

The effect of resource reduction was severe for both pollen and nectar reduction levels above 20%. Though these effects were independent of each other, pollen reduction had a slightly more severe impact on reproductive output than nectar reduction (Fig. 3). Reductions in new brood (first and second broods together) greater than 10% corresponding to a sublethal effect on the queen’s egg-laying rate resulted in severe declines in reproductive output. Also apparent reductions in the first brood due to exposure exacerbated the effects seen by a reduction in the second brood (workers that emerge on day 35) in Fig. 4. We emphasize this is an effect of fewer workers produced by the queen as opposed to any lethal exposure of workers to a pesticide.

### Lethal and sublethal effects combined

**Fig. 5:**
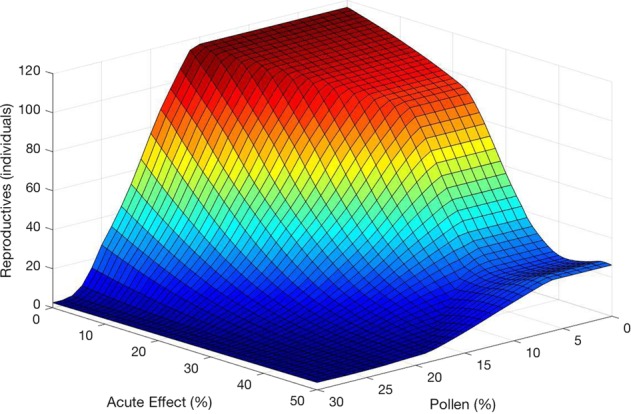
Effects of combined lethal (“Acute”) and sublethal (“Pollen” reduction) toxic insults on bumble bee reproductive output

Simulations of combinations of lethal and sublethal effects resulted in a non-linear interaction, demonstrating a synergistic effect. Declines in reproductives occurred after approx. 30% reductions solely due to lethal effects, or 20% solely in pollen reductions; the combination of these two levels resulted in nearly double the decline of reproductives (Fig. 5).

## Discussion

The Millennium Ecosystem Assessment (2005) provided a conceptual framework for linking environmental health and human well-being; protection of ecosystem services such as biocontrol and crop pollination are central themes. In the past decade, significant efforts aimed at better understanding the effects of toxicants such as pesticides on hymenoptera—especially honeybees—have been made (Lundin et al. 2015). Although empirical studies on the effects of toxicants on non-*Apis* hymenoptera are increasing (e.g., Rundlöf et al. 2015; Stanley et al. 2016), assuming that our knowledge of one species’ responses can be applied directly to other species risks creating confusion and misunderstandings (Banks et al. 2014). Recent physiological studies have corroborated this, demonstrating that pesticides such as pyrethroids affect honeybees (*A. melifera*) and bumble bees (*B. terrestris*) in fundamentally different ways (e.g., Kadala et al. 2019). Assessment and maintenance of the protection of ecosystem services relies fundamentally on a deep understanding of population dynamics; both empirical and theoretical approaches are important tools in this effort. Explorations of bumble bee population dynamics that incorporate our understanding of biological processes with predictive mathematical models provide a powerful means of prescribing protective measures and best practices. Here we have used a mechanistic model tailored to bumble bee colony development in an attempt to better understanding the response of bumble bees to toxicants such as pesticides. Our use of a delay differential equation model enables us to explicitly describe the effect of larval incubation and colony history on population outcomes. This level of detail allows us to demonstrate the sensitivity of colony viability to the timing and severity of pesticide sprays. Furthermore, the DDE model requires far fewer parameter estimations than approaches that use agent-based or individual-based models (e.g., Becher et al. 2014,2018). Empirical efforts that track real-time survivorship and behavior of larvae, workers and queens over a longer time period (similar to those conducted by Crall et al. (2015, 2018) but extended to larvae and for longer time periods would be useful for validating the DDE model presented here.

Understanding the mechanisms underlying the effects of resource availability on bumble bee population growth is an increasing focus of field and theoretical studies (Winfree et al. 2009; Williams et al. 2012); a recent study by Crone and Williams (2016) illustrates the importance of parsing out the relative importance of putatively important drivers (e.g., colony growth rates and floral resource availability) of bumble bee population outcomes. Less is known about combinations of reduced resource provisioning and diminished survivorship that may result from exposure to pesticides or mixtures of pesticides that have both lethal and sublethal effects, though the potential for multiplicative effects have been demonstrated in recent elegant experiments (e.g., Gill et al. 2012). Our simulations suggest that, even at low levels, sublethal effects such as reduced pollen foraging ability may result in severe declines in reproductive output if combined with lethal effects over 40%, for instance (see Fig. 5). This underscores the importance of better understanding the effects of exposure to mixtures of toxicants.

In the current analysis, our model highlights several important aspects pertaining to population implications of pesticide exposure in bumble bees. First, the overall impact of acute effects such as those exhibited by an LC_50_ or LD_50_ dose varies greatly with timing of exposure, with pesticide applications later in the development of the colony having relatively little effect compared with applications imposed within the first 30 days (Fig. 2). The immediate reduction in workforce size prevents the same level of foraging as seen before pesticide exposure, thereby limiting the resources available to rear future broods. In addition, the reduced number of workers also results in neglected larvae which ultimately limits future brood sizes and further impacts the production of reproductive bees. Perry et al. (2015) similarly found that early reductions in foraging ability in honeybees could have dramatic impacts at the population level later on; they suggest that these types of delayed responses due to early stressors may help explain field observations and experiments documenting colony collapse disorder. Our model results likewise suggest that delays in pesticide applications could significantly lessen deleterious effects on bumble bee populations.

Second, sublethal effects on the population output due to reduced egg-laying rates may be lessened if reductions are kept below 10%. However, higher levels of reduced egg-laying rates in the first brood may interact synergistically with subsequent brood exposure (due to repeated exposure to the same pesticide, or exposure to another, different chemical), wreaking havoc on the population at higher levels even for low levels of reductions on the second brood (Fig. 4). Field studies exploring combinations of pesticides on bumble bee colony outcomes have revealed similar effects (e.g., Gill et al. 2012). Because bees in farmland mosaics are often exposed to multiple spray events, sometimes with multiple pesticides, these types of knock-on effects may be difficult to mitigate in practice.

The synergistic effects revealed in our simulations emphasize the need to carefully consider population endpoints when gauging risk to bumble bees from pesticides and other toxicants; none of these effects would be detectable from simple LC_50_ analyses. Taken together, our results suggest that more sophisticated mathematical treatments of population processes are critical for assessing mechanisms underlying the effects of pesticides on bumble bees. Particular attention should be paid to timing of pesticide exposure, as well as the specifics of combinations of pesticides to which bumble bee colonies might be exposed. Finally, empirical data should be generated to test and validate the specific outcomes predicted by the model.

## Supplementary Information

Appendix
