# Supplementary material for: Lethal and sublethal effects of toxicants on bumble bee populations: a modelling approach
Source: Ecotoxicology. 2020 Feb 14;29(3):237–45. doi: 10.1007/s10646-020-02162-y (PMC7103009; doi:10.1007/s10646-020-02162-y)
Supplement: Supplementary file 1 — Appendix [file 10646_2020_2162_MOESM1_ESM.pdf]

Model

Resources:

$$\frac{dN}{dt} = (b_{NW} - \mu_{NW})W - 2[c_1(L_1^{(w)} + L_1^{(m)} + L_1^{(g)}) + c_2 L_2^{(w)} + c_3 L_2^{(m)} + c_4 L_2^{(g)} + c_5 L_3^{(g)}] \tag{1}$$

$$\frac{dP}{dt} = (b_{PW} - \mu_{PW})W - [c_1(L_1^{(w)} + L_1^{(m)} + L_1^{(g)}) + c_2 L_2^{(w)} + c_3 L_2^{(m)} + c_4 L_2^{(g)} + c_5 L_3^{(g)}] \tag{2}$$

Workers:

$$\frac{dW}{dt} = b_W^*(t-22)\exp[\Phi^{(w)}(t-18) - \Phi^{(w)}(t-9)] - \mu_W W \tag{3}$$

$$\frac{dL_1^{(w)}}{dt} = b_W^*(t-4) - \mu^{(w)}(t)\ L_1^{(w)} W - b_W^*(t-10)\exp[\Phi^{(w)}(t-6) - \Phi^{(w)}(t)] \tag{4}$$

$$\frac{dL_2^{(w)}}{dt} = b_W^*(t-10)\exp[\Phi^{(w)}(t-6) - \Phi^{(w)}(t)] - \mu^{(w)}(t)\ L_2^{(w)} W - b_W^*(t-13)\exp[\Phi^{(w)}(t-9) - \Phi^{(w)}(t)] \tag{5}$$

Males:

$$\frac{dM}{dt} = b_M^*(t-26)\exp[\Phi^{(m)}(t-22) - \Phi^{(m)}(t-11)] \tag{6}$$

$$\frac{dL_1^{(m)}}{dt} = b_M^*(t-4) - \mu^{(m)}(t)\ L_1^{(m)} W - b_M^*(t-12)\exp[\Phi^{(m)}(t-8) - \Phi^{(m)}(t)] \tag{7}$$

$$\frac{dL_2^{(m)}}{dt} = b_M^*(t-12)\exp[\Phi^{(m)}(t-8) - \Phi^{(m)}(t)] - \mu^{(m)}(t)\ L_2^{(m)} W - b_M^*(t-15)\exp[\Phi^{(m)}(t-11) - \Phi^{(m)}(t)] \tag{8}$$

Gynes:

$$\frac{dG}{dt} = b_G^*(t-30)\exp[\Phi^{(g)}(t-26) - \Phi^{(g)}(t-13)] \tag{9}$$

$$\frac{dL_1^{(g)}}{dt} = b_G^*(t-4) - \mu^{(g)}(t)\ L_1^{(g)} W - b_G^*(t-10)\exp[\Phi^{(g)}(t-6) - \Phi^{(g)}(t)] \tag{10}$$

$$\frac{dL_2^{(g)}}{dt} = b_G^*(t-10)\exp[\Phi^{(g)}(t-6) - \Phi^{(g)}(t)] - \mu^{(g)}(t)\ L_2^{(g)} W - b_G^*(t-13)\exp[\Phi^{(g)}(t-9) - \Phi^{(g)}(t)] \tag{11}$$

$$\frac{dL_3^{(g)}}{dt} = b_G^*(t-13)\exp[\Phi^{(g)}(t-9) - \Phi^{(g)}(t)] - \mu^{(g)}(t)\ L_3^{(g)} W - b_G^*(t-17)\exp[\Phi^{(g)}(t-13) - \Phi^{(g)}(t)] \tag{12}$$

Larval Mortality:

$$\frac{d\Phi^{(k)}}{dt} = \mu^{(k)}(t)W(t), \qquad \text{where index } k \text{ denotes class w, m, or g} \tag{13}$$

For $\theta \in [T_s - 8, T_s + 22]$,

$$\begin{aligned} &N(\theta) = N_0 && W(\theta) = R(W_0). \\ &P(\theta) = P_0 && L_1^{(w)}(\theta) = R(L_0^{(W)}) \\ &M(\theta) = 0 && L_2^{(w)}(\theta) = 0 \\ &L_1^{(m)}(\theta) = 0 && G(\theta) = 0 \\ &L_2^{(m)}(\theta) = 0 && L_1^{(g)}(\theta) = 0 \\ &L_2^{(g)}(\theta) = 0 && L_3^{(g)}(\theta) = 0 \\ &\Phi^{(k)}(\theta) = 0, \end{aligned} \tag{14}$$

where k indicates class w,m, or g and $R()$ is a ramp function.

A) SETUP

A bumblebee colony is modeled over a single season using a system of delay differential equations (DDE's). The model includes 12 variables for resources and different classes of bees, including in-nest nectar (N), in-nest pollen, (P), workers (W), males (M), gynes (G) and their respective larvae which are subdivided into age groups: worker larvae is two-stage ($L_1^{(w)}$, $L_2^{(w)}$), male larvae is two-stage ($L_1^{(m)}$, $L_2^{(m)}$), and gyne larvae is three-stage ($L_1^{(g)}$, $L_2^{(g)}$, $L_3^{(g)}$). These variables are described in (1) - (12). The model captures the behavior of a colony from its initiation in the spring (T_s) until hive functions cease in winter (T_w). We note that with limited information in the literature regarding the behavior of queens prior to the emergence of the first brood, our simulations begin at time $T_s + 22$ when the first workers begin assisting in colony activities. This is observed in the necessary history functions of model defined on the time domain $\phi \in [T_s - 4, T_s + 22]$ in (14). This choice reflects the longest fixed delay in the model of 26 days which represents the post egg maturation time of gynes, see table 2. It is important to note that this model describes a colony with a late switch time defined by the day $T^* = 40$ where the first male and gyne eggs are introduced into the hive and on day $T^{**} = 44$ when the last worker eggs of the hive are laid (Duchateau & Velthuis,1988).

B) RESOURCES

The primary sources of nourishment in a bumblebee colony include in-nest stores of pollen and nectar. Once the first workers emerge, some members commence foraging for the resources (b_{NW},b_{PW}) while others act as caregivers, remaining in the hive to distribute resources to the larvae and consume stored resources (μ_{NW}, μ_{PW}). Larval consumption is the primary draw from the stored resources where nectar is consumed at twice the rate of pollen (Pereboom,2000). Larvae of different subclasses grow at different rates which is directly related to the amount of resources that are consumed at each stage (Ribeiro,1994). This is reflected in the five different consumption parameters, c_i's as seen in expression $c_1(L_1^{(w)} + L_1^{(m)} + L_1^{(g)}) + c_2 L_2^{(w)} + c_3 L_2^{(m)} + c_4 L_2^{(g)} + c_5 L_3^{(g)}$ from (1) and (2). Note the first stage of each subclass is defined such that their members share the same consumption rate but members of $L_1^{(w)}$, $L_1^{(m)}$, and $L_1^{(g)}$ consist of different age groups. Consumption of stored resources by the queen was considered but due to a lack of quantifying information for queen behavior in the literature this mechanism was omitted in carrying out the simulations.

C) LARVAL DYNAMICS

The driving force of the model is larval population control in the presence (or lack thereof) of resources and worker caregivers. Bumblebees develop over a series of life stages for which we have made certain assumptions in the model. Eggs are introduced into the hive at continuous time dependent rates ($b_w(t)$, $b_m(t)$, $b_g(t)$), require minimal care from the workers, and under the stress of resource limitation are removed by

oophagy thereby decreasing the size of the brood during it's larval phase. During the larval phase, workers care for and feed juveniles under normal conditions. When the hive is under stress either resource limitation or insufficient worker population, larval ejection can occur as an additional population control on juveniles (Roger et al., 2017,). Each of the juvenile phases have fixed duration in the model and once a juvenile pupates it will emerge as an adult after the fixed time delay, see table 2.

We implement a system of delay differential equations (DDE's) in our model which can quantify the cumulative effect of larval ejection on a brood over their larval lifetime. To calculate the cumulative effect of larval ejection, ejection rates of larvae per worker, $\mu^{(k)}(t)$ (where k indicates worker, male or gyne class), can be integrated over fixed bounds in the delay differential equation (13), with a delay of zero days $(t-0)$. One can think of the additional state variables ($\Phi^{(w)}$, $\Phi^{(m)}$, $\Phi^{(g)}$) as cumulative rates of decay due to larval ejection efforts. The size of a brood can only decrease after all eggs have been laid, so an expression such as $\exp[\Phi^{(m)}(t-8)-\Phi^{(m)}(t)]$ (see (8)) represents the proportion of decay over the previous 8 days by larval ejection. We multiply this expression by the number of male eggs that entered the larval state 8 days ago, $b^*_M(t-12)$, to determine how many male larvae are entering the second male larvae state $L_2^{(m)}$ in (8). By considering table 2, we see that at a fixed time male juveniles would be entering state $L_2^{(m)}$ after 12 days given they had not been ejected during the prior 8 day period in the $L_1^{(m)}$ state. Here delays in states $\Phi^{(w)}$, $\Phi^{(m)}$, and $\Phi^{(g)}$ allow the model to compute the survival of individual broods (defined by the day their eggs were laid) without having to track them explicitly. This functionality makes delayed differential equations a more appropriate method for this system as opposed to ordinary differential equations.

D) OOPHAGY

Oophagy is the process of worker and queen bumblebees eating eggs that have been laid before they turn into larvae. This mechanism is not well studied but has been observed in relation to bumblebee pollen diets, e.g., low pollen quality or low amounts of protein available for the hive correlated with high amounts of oophagy (Genisse et al., 2002). By defining the number of resources that existing larvae want to consume as

$$C = c_1(L_1^{(w)} + L_1^{(m)} + L_1^{(g)}) + c_2 L_2^{(w)} + c_3 L_2^{(m)} + c_4 L_2^{(g)} + c_5 L_3^{(g)}, \tag{15}$$

we recognize this as the larval consumption term for our resources in (1) and (2). It follows from P being the available amount of in-nest pollen that a deficiency in pollen would be represented by positive values of the expression $C-P$. To determine the severity of the deficiency, we can use the expression:

$$\frac{C-P}{C+\epsilon} = \frac{\text{deficienty}}{\text{consumption}} \tag{16}$$

We expect high oophagy (or larval ejection as discussed later) when the expression is close to 1 and none when it's negative. We do not use oophagy directly in the model but instead scale the egg laying rates ($b_W(t)$,$b_M(t)$,$b_G(t)$) within corresponding functions b^*_W,b^*_M, and b^*_G defined as follows:

$$b^*_k(t) = b_k(t)\left(1 - \max\left[\frac{C(t)-P(t)}{C(t)+\epsilon}, 0\right]\right). \tag{17}$$

where k denotes the worker, male or gyne class of bumblebee. In (16) and (17), ϵ is a small correction that ensures the expression is well defined when no larvae are in colony implying $C=0$.

E) LARVAL EJECTION

Larval Ejection is a biological mechanism in bumblebee colonies which can control the number of larvae present. This behavior is not well studied; we propose this behavior could occur when not enough workers are present to care for the larvae and when the hive is under stress by a resource deficiency (Roger et al., 2017, Tasei & Aupinel, 2008, Pomeroy,1979, Tasei, Lerin, & Ripault, 2000). Under a resource deficiency, the model uses a mechanism similar to that for describing criteria for oophagy, see (16). In order to design a mechanism for neglect, consider that there exists an optimal larvae to worker ratio Z which represents

the number of larvae that can be cared for by each worker in the hive. The optimal sustainable larval population is ZW. It follows that the total larval population in the colony is the sum of its larval subclasses, $L = \sum_{i,k} L_i^{(k)}$ where $i = 1, 2, 3$ and k indicates class w,m, or g. Next, we define the excess larvae, those that cannot be cared for as $L - ZW$ and can create a scaling factor for neglect as follows:

$$\frac{L - ZW}{L + \epsilon} = \frac{\text{excess larvae}}{\text{total larvae}}. \tag{18}$$

This factor is low when $L < ZW$ and higher as L exceeds ZW (the maximum larvae that can be cared for). Putting these two scaling terms together with maximum larval ejection rates by workers (α and β), we produce an expression for larval ejection,

$$\mu^{(k)}(t) = \alpha \max\left[\frac{L - ZW}{L + \epsilon}, 0\right] + \beta \max\left[\frac{C - P}{C + \epsilon}, 0\right]. \tag{19}$$
